# 213. Do Patients with Candidemia Need an Ophthalmology Exam?

**DOI:** 10.1093/ofid/ofad500.286

**Published:** 2023-11-27

**Authors:** Alice Lehman, Katelyn Tessier, Victoria Sattarova, Sandra Rocio Montezuma, Susan E Kline, Serin Edwin Erayil

**Affiliations:** University of Minnesota, Minneapolis, Minnesota; University of Minnesota, Minneapolis, Minnesota; University of Minnesota, Minneapolis, Minnesota; University of Minnesota, Minneapolis, Minnesota; University of Minnesota Medical School, Minneapolis, Minnesota; University of Minnesota Medical School, Minneapolis, Minnesota

## Abstract

**Background:**

Candidemia can lead to hematogenous seeding of the eye resulting in chorioretinitis or endophthalmitis. IDSA recommends a screening dilated retinal examination for patients with candidemia, whilst American Academy of Ophthalmology (AAO) recently declared this a low value practice. Using an inter-disciplinary team, we evaluated ophthalmology examination for patients with candidemia.

**Methods:**

We performed a retrospective study of patients with candidemia at an academic medical center and affiliated hospitals between January 2011 – June 2022. Electronic medical records were reviewed for demographics and baseline characteristics. The primary outcome was chorioretinitis and/or endophthalmitis. The study was adjudicated by independent review from infectious disease and ophthalmology providers.

**Results:**

We included 308 patients with a blood culture positive for *candida* species. 149 patients (48%) received an ophthalmologic exam, of which 55% (82) were asymptomatic. Abnormal ocular findings concerning for presumed ocular candida complications were found in 12 patients (8%, 12/149), of which 4 had alternative diagnoses to candida choriorentinits or endophthalmitis. All of the remaining 8 patients (5%, 8/149) had presumed candida chorioretinitis with no cases of candida endophthalmitis and this led to a change in management in 88% (7/8) of patients (Table 1). Of the 8 patients, 3 patients were asymptomatic, making the number needed to screen 28 to detect presumed candida chorioretinitis amongst asymptomatic patients. We did not identify statistically significant risk factors for development of abnormal ocular findings (Table 2).

Table 1

**Figure:**
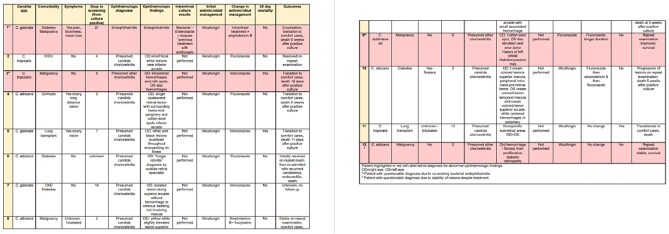

Clinical diagnosis and management of patients with candida chorioretinitis and/or endophthalmitis

Table 2

**Figure:**
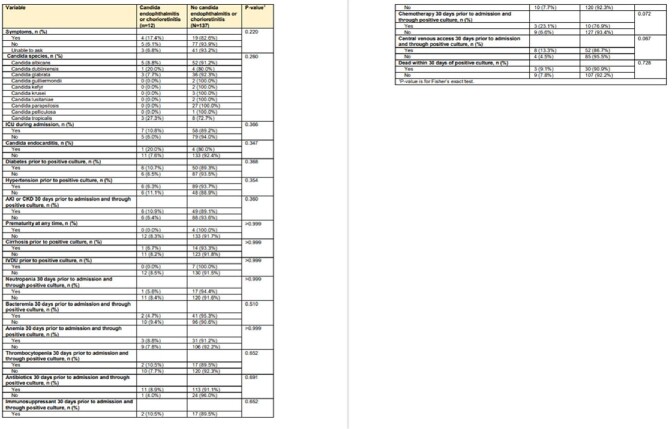

Clinical features of patients with and without ocular candidiasis

**Conclusion:**

IDSA and AAO recommend ophthalmologic examination in symptomatic and intubated patients with candidemia. We found a 5% incidence of presumed ocular candidiasis in patients with candidemia. Given the limitations of our study, we could not conclude for or against screening in asymptomatic patients as this did not lead to a significant change in patient outcomes. Inter-disciplinary decisions involving ophthalmology and infectious diseases can help inform best practices for screening in asymptomatic patients. Future studies should evaluate ocular photography and teleophthalmology as screening strategies.

**Disclosures:**

**All Authors**: No reported disclosures

